# Construction of Low-Carbon Ferry—A Case of Jingning, China

**DOI:** 10.3390/ijerph19116451

**Published:** 2022-05-26

**Authors:** Shiru Yao, Gengyong Cao, Zi Zhan, Qinqin Cao, Hailu Fu, Wenjie Dong

**Affiliations:** 1 School of Quality and Safety Engineering, China Jiliang University, Hangzhou 310018, China; shiruyao1999@126.com (S.Y.); zz942694@126.com (Z.Z.); cqq0723@126.com (Q.C.); 13a0604073@cjlu.edu.cn (H.F.); 2 Zhejiang Scientific Research Institute of Transport, Hangzhou 310012, China; caogengyong@126.com

**Keywords:** low carbon, ferry, carbon emission, carbon sink

## Abstract

As the Chinese government has pledged to reach its carbon peak by 2030 and carbon neutrality by 2060, it is necessary to investigate how regional sustainable development can be achieved. This paper used a ‘bottom-up’ model to calculate the ferry carbon emissions in Jingning, China, and proposed four measures to reduce carbon emissions, including renewing ferryboats, planting water-level-fluctuating zones, greening the ferries, and installing solar energy. Quantitative analyses were conducted to calculate the possible emissions reduction from 2021 to 2025, with the results indicating that the total emissions could be reduced by 392.67 t. Finally, a new low-carbon ferry concept is proposed, based on simultaneous carbon emission reduction and carbon sink enhancement. This study provided a theoretical and decision-making reference for the operation of green, beautiful, and low-carbon ferries.

## 1. Introduction

Greenhouse effects, such as global warming, glacial melting, sea-level rises, and haze, are endangering the future survival of the planet [1], which has aroused widespread international concerns [2]. Because carbon dioxide emissions are a key component of greenhouse gases, many countries have implemented carbon emissions peak and neutralization plans to mitigate the greenhouse effect. To realize a sustainable development path, the Chinese government has pledged that the country’s carbon emissions will peak before 2030, and carbon neutrality will be achieved before 2060, both of which will bring opportunities and challenges [3] to the energy and economic sectors. To achieve carbon neutralization, China has introduced various measures, such as controlling carbon dioxide emissions, increasing carbon sinks, developing key technologies, formulating policies, and strengthening publicity and education [4,5]. Consequently, carbon emission reduction activities have been implemented in regions around the country.

Jingning is an autonomous county in China; it is located in the central mountainous area of southern Zhejiang province. Because of its complex terrain, the development of road transportation has been restricted. Therefore, Jingning villagers have always traveled by ferryboat. The county has five towns, 71 administrative villages, 416 natural villages, and a permanent resident population of 42,000. Besides, Jingning county is famous for tourism, with the total tourism revenue in 2020 reaching CNY 6.746 billion. The travel demands of residents and the tourism industry have meant higher requirements for the county’s ferry transportation.

Research has tended to focus on port emissions reduction or sustainable port developments, rather than ferry construction processes [6]. Measures introduced to reduce port emissions have included improving handling efficiencies, replacing heavy fuel oil with low-sulfur fuel oil and shore power, using renewable energy, and implementing energy efficiency measures [7,8]. Ship emissions reduction measures have included energy greening, route optimization, automated mooring, and speed reductions [9,10,11]. The carbon emissions operations and management for ferries and ports are different. However, there have been few studies on ferry developments. As the capital investment required to implement port emission reduction measures is often too high to realize in practice for ferries [12], it has become imperative to explore the possibilities of sustainable ferry development.

This paper evaluated the ferryboat carbon emissions in Jingning county. It proposed green ferry development strategies to reduce carbon emissions and increase carbon sinks, which provide a theoretical support and rational decision-making basis for ferry reconstruction and emissions reductions in other regions.

## 2. Materials and Methods

### 2.1. Carbon Dioxide Emission Calculation Method for Ferryboats

According to the carbon dioxide accounting methods, carbon dioxide emission calculation can be divided into ‘top-down’ and ‘bottom-up’ methods. The former starts from the country’s total consumption and is decomposed into various industries for accounting. The latter is carried out by measuring the energy consumption of subordinate units. Considering the data availability, we choose the ‘bottom-up’ method [9,13]. First, the refueling volumes of the ferryboats in 2021 were obtained through field research and converted into the standard coal equivalent. Then, the annual carbon emissions were calculated based on the standard coal equivalent and carbon emissions factors.

In Formula (1), the standard coal equivalent coefficient of 1.4571 kg standard coal/kg was extracted from the *General Rules for the Calculation of Comprehensive Energy Consumption* (GB/T 2589-2008). Additionally, the carbon emission factor of 1.76 t/MkJ was extracted from the *Guidelines for the Preparation of Provincial Greenhouse Gas Inventories*: (NDRC Climate [2011] No. 1041). The ferryboats’ data were provided by the Jingning County Rural Ferry Bus Co., Ltd. (Lishui, China).

CO_2_ emisssions = F × α × β (1)

F: refueling volume of ferryboats, t; α: equivalent standard coal coefficient, 1.4571 kg standard coal/kg; β: carbon emissions factor, 1.76 t/MkJ.

### 2.2. Carbon Reserve Estimation Method in Water-Level-Fluctuating Zones

As trees exchange CO_2_ and O_2_ with the environment through photosynthesis, planting a water-tolerant tree species in the water-level-fluctuating zone can convert CO_2_ into organic matters with solar energy and act as a carbon sink [14]. However, it is necessary to determine the diurnal tree photosynthesis capacities, carbon sequestration per leaf area, and carbon sequestration per unit of land area, which varies from year to year [15]. Taxodiaceae (swamp cypress) has strong water adaptability and high landscape value. The diameter of a mature Taxodiaceae root can reach more than 10 m, and it has a good soil fixation ability. It can also provide a suitable habitat for animals and protect biodiversity. Based on these advantages, the Taxodiaceae was planted in the water-level-fluctuation zone. Table 1 shows the biomass and carbon storage of Taxodiaceae in different years.

After determining the carbon storage of the trees in different years, the water-level-fluctuating zone’s ferry carbon storage was calculated using Formula (2):

(2) $${\mathrm{CO}}_{2\;}\;\mathrm{reserves}\;=\;R\;\times \;S$$

R: carbon storage, t/ha; S: area, ha.

### 2.3. Carbon Sequestration Calculation Method for Green Areas

Carbon sequestration from lawns and shrubs can be estimated by unit biomass, whereas the carbon sequestration of trees needs to be estimated based on trunk size [17]. The unit biomass in Formula (3) was derived from the *IPCC Grassland Biomass Reference Value*, and the unit accumulation and BEF in Formula (4) were taken from the *IPCC Biomass Transformation and Expansion Factor Reference Value*, both of which are internationally recognized coefficients. The unit biomasses for lawns and shrubs were 14 and 9.8 t/ha, unit tree biomass was 20 m^3^/ha, tree density was 0.45 t/m^3^, BEF was 6, and biotransformation coefficient was 0.5.

The carbon sequestration calculation formula for lawns and shrubs was as follows:

CO_2_ reserves = B × S × θ × γ (3)

B: unit biomass, ton/ha; S: area, ha; θ: biotransformation coefficient; γ: M_CO__2_/M_C._

The carbon sequestration calculation formula for the trees was as follows:

CO_2_ reserves = V × S × ρ × BEF × θ × γ (4)

V: unit accumulation, 20 m^3^/ha; ρ: unit biomass of trees, 0.45 t/m^3^; BEF: biomass expansion factor.

### 2.4. Carbon Reduction Calculation Method for Lamps

Using solar lights on the ferry can save electricity from fossil fuel combustion. The carbon dioxide emissions reductions from using solar lamps were calculated using Formula (5), with the carbon emissions coefficient in the formula based on the *2019 Emission Reduction Project China Regional Grid Baseline Emissions Factor*. Zhejiang Province is part of the East China power grid, for which the carbon emissions coefficient is 0.5246 t CO_2_/MWh.

CO_2_ reduce = P × n × T × δ × 365 (5)

P: power, kW; N: number of lights; T: time, h; δ: carbon emission index, t CO_2_/MWh.

## 3. Results

### 3.1. Carbon Emission Accounting of Ferryboats in 2021

**Figure 1:**
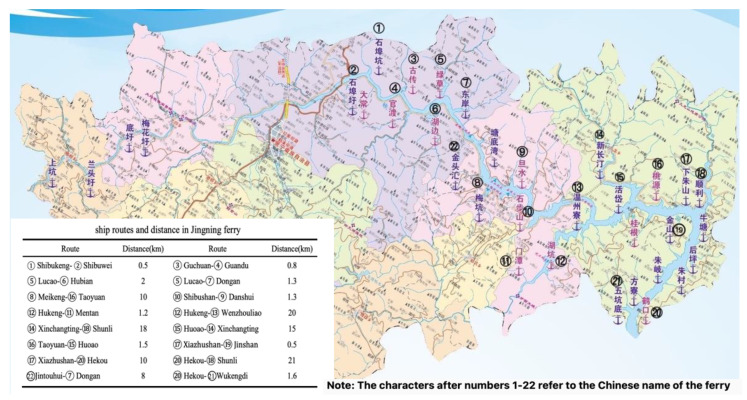
Jingning county ferry distribution map.

Figure 1 shows the ferry distribution in Jingning County. Ferries operate on two sides of Qianxia Lake in the main population areas, with the ferry routes established to transfer personnel and vehicles. As shown in Figure 1, there are 16 shipping lines, and, except for the Guchuan Ferry to Guandu Ferry, which has both passenger and vessel ferryboats, the remainder only has one kind of ferryboat going back and forth. In addition, bridges have been built in areas where there is less population and short-distance ferries.

Table 2 gives the shipping data for Jingning county in 2021, at which time there were 17 motorized and 4 non-motorized ferryboats. The motorized ferryboats were traditional all-diesel ferryboats, each of which had a specified route (the 17 ferryboats in Table 2 correspond to the 16 routes in Figure 1) and fixed departure times. The ferryboats’ power, route distances, and total weights all affect fuel consumption, the annual fuel consumption for which was determined based on their refueling data. The annual carbon emissions for each ferryboat were calculated using Formula (1). The aggregate vessel emissions in Jingning County in 2021 were 368.06 t, with the passenger ferryboat carbon emissions being 176.03 t or 47.83% of the total; the total carbon emissions for the vehicle ferryboats was 192.03 t or 52.17%.

### 3.2. Emission Reduction Measures and Effects

#### 3.2.1. Renewal of Ferryboats Electrification

Ferryboats are the largest source of carbon emissions in ferries; conventional ferryboats use diesel engines/generators to spray carbon dioxide and harmful particles into the atmosphere, and ferryboat electrification and renewal are needed to achieve the low-carbon goals [18,19]. The electrification of the ferryboats requires onboard electrical storage equipment and a shore power network [20]. In Jingning, the existing passenger ferryboats are gradually being converted into electric. Moreover, shore power is an efficient way to provide energy for ferryboats and reduce carbon emissions. However, electric ferryboats have a high cost, short endurance, small battery capacity, power source degradation, and charging difficulty problems [21]. As the shipping routes in Jingning County are relatively short and the endurance requirements are low, establishing several shore-side charging stations can overcome the charging problems.

Jingning has already developed a project plan for the electrification of its ferryboats. Table 3 shows the data obtained by statistics. From 2021 to 2025, it plans to upgrade two electric passenger ferryboats, which is estimated to reduce carbon dioxide emissions by about 32 t per year; by 2028, it plans to have upgraded all 11 passenger ferryboats. The current battery life is generally about five years; therefore, the associated battery costs will be for battery replacements and maintenance. In 2025, the annual energy costs for the electrified ferryboats are expected to be around CNY 480,000, and the battery costs are expected to be CNY 200,000. The electrification of ferryboats can reduce carbon emissions by reducing diesel consumption. It has the advantages of higher safety, lower use cost, and no diesel leakage. Ferryboats electrification is a realistic choice for the green transformation of ferries.

#### 3.2.2. Construction of Water-Level-Fluctuating Zones

The drawdown zone of the ferry is commonly called the ‘water-level-fluctuating zone’ or the ‘hydro-fluctuation belt’ [22]; it has also been called the ‘littoral zone’, because it is somewhat analogous to seashore tidal zones. The water-level-fluctuating zone is an essential part of a river landscape. Reasonable planting in the area can increase the integrity and aesthetics of the zoning landscape, stabilize the riverbanks, purify the water quality, protect the biodiversity, beautify the environment, and act as a natural carbon sink [23]. Methods have been proposed for the development and utilization of water-level-fluctuation zones in China. However, many are challenging to directly apply, due to the differences in the water level fluctuation durations and natural conditions, such as the climate, topography, and soil texture. Therefore, based on the rapid fluctuation and sizeable seasonal water level differences in Jingning, the water-resistant Taxodiaceae (swamp cypress) was selected for the water-level-fluctuation zone.

In 2021, 3000 trees were planted in the water-level-fluctuation zone, which was made up of Taxodium ‘Zhongshansha 302’, Taxodium ascendens, and Metasequoia glyptostroboides. In 2022, locals plan to plant 1500 trees, after which, 1000 trees a year will be planted from 2023 to 2025. The trees are all only about 1-year-old and are planted at intervals of 1.5 m × 1.5 m, covering a total area of 1.688 hectares. As calculated using Formula (3), the 7500 young trees will absorb 38.45 t of carbon dioxide every year by the end of 2025. All the young trees will grow into mature forests by the end of 2050; from 2021 to 2050, the water-level-fluctuating zone is expected to store 577.33 t of carbon dioxide. The construction of water-level-fluctuation zones can improve the carbon sink capacity of the ferry and create a stable natural wetland ecosystem, which can enrich biodiversity.

#### 3.2.3. Construction of Lawn-Type Green Areas

Lawns cover a significant part of the green open urban and rural greening spaces (up to 70–75%) [24] and have social, ecological, and aesthetic value. Building lawn-type green spaces can also dramatically improve carbon sink capacity. Depending on each ferry’s situation, plans have been made to improve the ferry greening and reform the ground’s water permeability in the pier parking areas. From 2021 to 2025, 44,340 m^2^ will be converted to green land at more than ten ferry piers, such as the pier areas for the Huitou, Xinchangting, Taoyuan, Guigen, Hekou, Meikeng, Tangdiwan, Jintouhui, Guandu, Danshui, and Shibushan Ferries. Due to the cost and the subsequent maintenance costs, a lawn-type green area was selected, with the ratio of trees to shrubs and lawns being 0.3:0.4:1 (evergreen trees/deciduous trees = 1:3; evergreen shrubs/deciduous shrubs = 3:1) [25] and the planting areas, respectively, being 0.78, 1.04, and 2.61 hectares.

Formulas (3) and (4) were used to estimate the total carbon sequestration of the lawn-type green area at 163.16 t, of which, the respective carbon sequestration for the grassland, shrubs, and trees would be 77.47, 18.74, and 66.94 t. The specific values are shown in Table 4 and Table 5. There are also plans to afforest 3950 m^2^ on five bridges: the Xikou, Xikou No. 2, Huitou, Jinzhong, and Gaopanyang bridges, which together are expected to absorb about 10 t of carbon dioxide annually.

#### 3.2.4. Use of Green Energy

The average annual sunshine duration in Jingning is 1841 h; therefore, it is a class IV solar energy resource area. As clean energy, solar energy has no carbon emissions in the usage process. It has high initial investment costs and low ongoing operational costs. Therefore, the use of photovoltaic technology in the streetlights at the ferry terminals and for the beacon lanterns on the river can reduce carbon emissions. As the lighting systems consume significant ferry energy, all lighting systems at the waiting pavilions and on the ferries have energy-saving LED lights to realize the intelligent control of 100% of the centralized lighting; they can be easily adapted to higher electricity, energy, environmental, and economic development needs.

Each ferry has 200 W solar outdoor streetlights installed at a height of 8 m, with the distance between the lights being 10 m. In 2021–2025, more than ten ferries are to be equipped with 332 solar lights, which, compared with traditional lights, is expected to reduce carbon dioxide emissions by 101.71 t annually. Table 6 shows the emission reduction of each ferry. Solar lights bring night lighting to the ferry without producing carbon dioxide and have the advantages of low maintenance costs and high safety performance.

#### 3.2.5. Low-Carbon Education and Publicity

Culture is an essential part of low-carbon ferry construction, as the involved departments need to cooperate to integrate their resources, coordinate the planning, and promote low-carbon knowledge [26]. Jingning is an ethnic enclave in eastern China, and it is famous for its cultural heritage and strong ethnic characteristics. In recent years, Jingning has given full play to its cultural advantages and developed a thriving tourist industry. Therefore, the government needs to simultaneously take the low-carbon concept as part of its tourism economy development and promote the low-carbon culture to the general public. However, promoting low-carbon tourism development requires a conceptual change [27]. First, the government needs to design a net zero-carbon tourism route with regional characteristics. Second, give full play to publicizing the low-carbon tourism routes and ferry construction through an app. Next, the department needs to conduct low-carbon activities, so tourists can experience low-carbon tourism in practice. Finally, the demonstration of practice cases shows the area’s low-carbon characteristics and encourages tourism development.

#### 3.2.6. Low-Carbon Ferry

**Figure 2:**
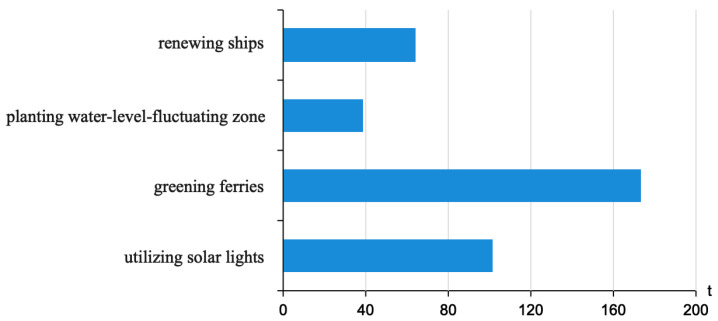
Cumulative emissions reductions in 2025.

As the infrastructure construction is expected to be completed in 2025, it is estimated that the total carbon emissions reduction from the ferryboat renewal, planting of the water-level-fluctuating zone, ferry greening, and solar lights will be 377.32 t. The lawn-type green space development is expected to have the most significant carbon emissions reduction, at 173.16 t or 45.9% of the total carbon reduction. The specific data is shown in Figure 2. In 2028, all 11 passenger ferryboats will be electrified, reducing the carbon dioxide emissions by 176 t, compared to 2021; as the trees mature, the carbon sequestration in the water-level-fluctuation zone will increase significantly.

Through the above research, the concept of the low-carbon ferry has been formed. Low-carbon ferry construction needs to reduce carbon dioxide emissions from ferryboats, ferrying processes, and auxiliary facilities. Furthermore, the ferry needs to explore the more natural potential to maximize its carbon sink capacity. The upgraded ferry infrastructure needs to be integrated with the actual environment, in order to give full play to the unique advantages and promote the development of a low-carbon regional tourism economy.

## 4. Conclusions

This paper evaluated the carbon emissions in Jingning County. It is pointed out that, in order to realize low-carbon development, Jingning needs more innovation to release more potential for natural carbon sequestration. Four measures to reduce carbon emissions were proposed:

- Ferry electrification and renewal;
- Water-level-fluctuating zone construction;
- Lawn-type green space construction;
- The rational use of green energy.

Besides these four measures, it is recommended that low-carbon publicity and education be intensified. Combining low-carbon culture with tourism could boost Jingning’s economy and ensure sustainable development. This research has formed a complete low-carbon ferry construction system for a small ferry, with the results providing valuable insights and guidance to ferry builders for green, low-carbon management practices. It could be a sound decision-making basis for ferry reconstruction and emissions reductions in other regions. More importantly, this research supports China’s carbon peak and carbon neutrality policies and promotes green high-quality development in Jingning county.

## Figures and Tables

**Table 1:** Biomass and carbon storage of Taxodiaceae in different years [16].

	Whole Tree Biomass (t/ha)	Carbon Storage (t/ha)
Young tree (6 years)	43.76	22.75
Middle-aged tree (11 years)	88.34	45.94
Nearly ripe forest (20 years)	148.20	77.06
Mature forest (25 years)	172.08	89.48
Over-ripe forest (33 years)	246.59	128.23

**Table 2:** The 2021 ferryboat carbon emissions statistics.

No.	Type	Weight (t)	Power (kW)	Fuel Consumption (t)	CO_2_ Emissions (t)
1	P	20	24	0.50	1.27
2	P	20	24	0.31	0.80
3	P	20	24	6.21	15.92
4	P	20	24	4.97	12.74
5	P	20	24	12.42	31.84
6	P	20	24	6.21	15.92
7	P	20	24	9.31	23.88
8	P	20	24	11.17	28.66
9	P	20	24	13.04	33.43
10	P	20	24	0.31	0.80
11	P	51	125	4.20	10.78
12	V	170	77	17.83	45.72
13	V	170	77	7.13	18.29
14	V	170	77	11.59	29.72
15	V	170	77	10.70	27.43
16	V	170	77	14.26	36.58
17	V	170	77	13.37	34.29
Total			-	143.52	368.06

Note: P means passenger ferryboats, V: means vessel ferryboat. Vessel ferryboats transfer cars and e-bikes.

**Table 3:** Passenger ferry electrification energy cost consumption.

Year	Type		Annual Fuel Consumption		Annual Operating Cost (Thousand CNY)	
	Diesel	Electric	Diesel (t)	Power Consumption (MkWh)	Energy Costs	Battery Costs
2021	11	0	69	0	412	0
2025	9	2	56	0.2	480	200
2028	0	11	0	1.1	770	1100

Note: Some of the electricity needed for ferryboat charging can be provided by photovoltaic panels.

**Table 4:** Carbon sequestration calculation of lawn and shrub.

	Unit Biomass (t/ha)	Area (ha)	Total Biomass (t)	Biotransformation Coefficient	Carbon Sequestration (t)
lawn	14	2.61	36.51	0.5	66.94
shrub	9.8	1.04	10.22	0.5	18.74

**Table 5:** Carbon sequestration calculation of tree.

	Unit Accumulation (m^3^/ha)	Area (ha)	Unit Biomass (t/m^3^)	BEF	Biotransformation Coefficient	Carbon Sequestration (t)
tree	20	0.78	0.45	6	0.5	77.47

**Table 6:** Emissions reduction from solar light installation on the ferries.

No.	Name	Quantity	Time (h)	Electricity Consumption (MWh)	Emission Reduction (t)
1	Huitou Ferry	20	8	0.03	6.13
2	Xinchangting Ferry	28	8	0.04	8.58
3	Taoyuan Ferry	85	8	0.14	26.04
4	Guigen Ferry	42	8	0.07	12.87
5	Hekou Ferry	32	8	0.05	9.80
6	Meikeng Ferry	6	8	0.01	1.84
7	Tangdiwan Ferry	22	8	0.04	6.74
8	Jintouhui Ferry	23	8	0.04	7.05
9	Guandu Ferry	23	8	0.04	7.05
10	Danshui, Shibushan Ferry	51	8	0.08	15.62
Total		332			101.71

## Data Availability

Not applicable.
